# Gametocyte carriage after seasonal malaria chemoprevention in *Plasmodium falciparum* infected asymptomatic children

**DOI:** 10.1186/s12936-021-03706-1

**Published:** 2021-03-26

**Authors:** Abdullahi Ahmad, Aurelia Prom, John Bradley, Mamadou Ndiath, Blessed Etoketim, Mamadou Bah, Jean-Pierre Van Geertruyden, Chris Drakeley, Teun Bousema, Jane Achan, Umberto D’Alessandro

**Affiliations:** 1 Disease Control and Elimination Theme, Medical Research Council Unit The Gambia At London, School of Hygiene and Tropical Medicine, P.O Box 273, Banjul, The Gambia; 2 MRC Statistics and Epidemiology Group, London School of Hygiene and Tropical Medicine, Keppel Street, London, WC1E 7HT UK; 3 Global Health Institute, University of Antwerp, Gouverneur Kinsbergencentrum, Campus Drie Eiken, Doornstraat 331, 2610 Wilrijk, Belgium; 4 Department of Immunology and Infection, School of Hygiene and Tropical Medicine, Keppel Street, London, WC1E 7HT UK; 5 Department of Medical Microbiology, Radboud University Medical Center, Geert Grooteplein 28, Microbiology 268, 6500 HB Nijmegen, The Netherlands

**Keywords:** Gametocyte carriage, Seasonal malaria chemoprevention, *Plasmodium falciparum*, Asymptomatic

## Abstract

**Background:**

Treatment of clinical *Plasmodium falciparum* malaria with sulfadoxine-pyrimethamine (SP) and amodiaquine (AQ) is associated with increased post-treatment gametocyte carriage. The effect of seasonal malaria chemoprevention (SMC) with SP and AQ on gametocyte carriage was assessed in asymptomatic *P. falciparum* infected children.

**Methods:**

The study was carried out in eastern Gambia. Asymptomatic *P. falciparum* malaria infected children aged 24–59 months old who were eligible to receive SMC (SMC group) and children 5–8 years that were not eligible to receive SMC (comparison group) were recruited. Gametocytaemia was determined by molecular methods before and after SMC administration. Gametocyte carriage between the groups was compared using the chi-squared test and within-person using conditional logistic regression.

**Results:**

During the 2017 and 2018 malaria transmission seasons, 65 and 75 children were recruited in the SMC and comparison groups, respectively. Before SMC administration, gametocyte prevalence was 10.7% (7/65) in the SMC group and 13.3% (10/75) in the comparison group (*p* = 0.64). At day 13 (IQR 12, 13) after SMC administration, this was 9.4% (5/53) in children who received at least the first dose of SMC treatment and 12.7% (9/71) for those in the comparison group (*p* = 0.57). Similarly, there was no difference in prevalence of gametocytes between children that adhered to all 3-day doses of SMC treatment 15.6% (5/32) and those in the comparison group (*p* = 0.68). In the SMC group, within-group gametocyte carriage was similar before and after SMC administration in children that received at least the first dose of SMC treatment (OR 0.6, 95% CI 0.14–2.51; *p* = 0.48) and in those that adhered to all 3-day doses of SMC treatment (OR 1.0, 95% CI 0.20–4.95; *p* = 1.0).

**Conclusion:**

In this study with relative low gametocyte prevalence prior to SMC treatment, no evidence was observed that SMC treatment increased gametocyte carriage in asymptomatic *P. falciparum* malaria infected children.

## Background

The global malaria burden has substantially reduced over the last two decades owing to the scale up of control interventions [1, 2]. In sub-Saharan Africa, *Plasmodium falciparum* is the predominant malaria species and remains the focus of control and elimination efforts [2]. Since 2012, the World Health Organization (WHO) recommends seasonal malaria chemoprevention (SMC) as an additional tool for malaria control where transmission is highly seasonal, primarily the Sahel region of sub-Saharan Africa [3]. SMC is the monthly administration of a full course of sulfadoxine-pyrimethamine (SP) and amodiaquine (AQ) to all children 3–59 months old during the 3–4 months of the malaria transmission season regardless of infection status with the goal of reducing malaria morbidity and mortality.

Given that many *P. falciparum* infections are asymptomatic in both high and low transmission settings [4, 5] and can persist for long periods of time [4, 6, 7], the systematic administration of anti-malarial drugs to the whole population or to high risk groups, such as children, results in the treatment of such infections. Most available anti-malarial treatments clear asexual parasite stages but with incomplete and variable effects on gametocytes [8], the sexual stages responsible for onward transmission to the vector. Treatment of symptomatic patients with SP, AQ, chloroquine (CQ), and piperaquine (PQ) is associated with increased gametocyte carriage and or density [9–14]. Mechanisms such as enhancement of gametocyte production in response to drug-induced stress, release of sequestered gametocytes and up-regulation of gametocyte production in response to subcurative dosage are current hypotheses for the emergence of post-treatment gametocytes [14, 15], that can be infectious to the vector [12–14, 16]. Although the transmission potential of post-treatment gametocytes has been known for about 100 years [17], it has only recently drawn attention in relation to the current drive towards malaria elimination and eradication and the increased knowledge on the differential impact of anti-malarials on gametocyte persistence and infectivity [18].

SMC consists of the monthly administration of SP and AQ over 3 days. Only the first day of treatment (SP and AQ) is directly observed by trained health workers; the other two daily doses of AQ alone are given to care givers to be administered at home, without supervision, which carries the risk of poor adherence [19, 20] and consequently sub-optimal drug levels in children already infected with malaria. Considering that both SP and AQ increase gametocyte carriage in *P. falciparum* malaria patients [10, 12] coupled with the risk of sub-optimal drug levels that could trigger gametocytogenesis [15], SMC may increase gametocyte carriage in asymptomatic malaria infected children, possibly resulting in onward transmission to the vector since it is administered during the rainy season when the malaria vector is abundant.

Here, the effect of SMC on post-treatment gametocyte carriage in asymptomatic *P. falciparum* infected children was assessed.

## Methods

### Study setting

This was a prospective study conducted during the 2017 and 2018 malaria transmission seasons in eastern Gambia. Malaria transmission in The Gambia, almost exclusively by *P. falciparum*, is highly seasonal, occurring mainly during the rainy season (July to October) and shortly after (November to December). Current control activities in the region include prompt diagnosis and treatment, vector control intervention such as indoor residual spraying (IRS) and insecticide-treated nets (ITN), and drug-based interventions such as intermittent preventive treatment during pregnancy (IPTp) and SMC, the latter implemented from 2014 onwards.

### Participants selection

Potential participants were identified through the health and demographic surveillance system (HDSS) data base of villages with relatively high malaria prevalence (≥ 20%) according to previous surveys [21]. These were children 24–59 months old and children 5–8 years old. The former represented the SMC group as they are eligible for SMC treatment; the latter were taken as comparison group as they do not receive SMC. Slightly older children were selected as a comparison group because all children within the SMC age group would receive the SMC treatment during the SMC campaigns. The assumption was that baseline gametocyte carriage would not vary between the two age groups [15, 22].

### Screening and enrolment

At screening, information on medical history was collected and a clinical examination was performed. A dried blood spot (DBS) was collected via a finger prick for the detection of *P. falciparum* infection by polymerase chain reaction (PCR). Children with asymptomatic *P. falciparum* infection, without history or evidence of chronic illness and who have not received anti-malarial treatment in the previous 2 weeks were enrolled.

### Study visits before and after SMC treatment

A visit was conducted by the study team close to the commencement of nation-wide SMC campaign (maximum 4 days prior). Demographic information and medical history, particularly any episode of clinical malaria or anti-malarial treatment since enrolment was collected. Axillary temperature and body weight were measured and recorded. Approximately 300µL of blood was collected by finger prick into an ethylene diamine tetra acetic acid (EDTA) microtainer tube for molecular detection and quantification of *P. falciparum* gametocytes. Haemoglobin was measured with a HemoCue® photometer (Ångelholm, Sweden). Parents and care givers were encouraged to participate in the upcoming SMC campaign and to ensure safe keeping of their SMC drug administration records.

The first SMC cycle was implemented by the Gambian National Malaria Control Programme. Briefly, a 3-day course of SP and AQ was administered by to all children aged 3 months to < 5 years in the communities. The dose for the first day (SP and AQ) was directly observed by the health worker while that of second and third days (AQ alone) was administered by the caregiver. An SMC distribution card to document all administered doses for every child was issued to care givers.

Study participants were then re-visited by the study team approximately 2 weeks after the administration of the first SMC cycle. This time interval was chosen to allow for the emergence of gametocytes in the peripheral circulation [23]. History of any episode of clinical malaria or anti-malarial treatment since first study visit was collected. Axillary temperature was measured and children with fever (body temperature > 37.5 ºC) were tested with a rapid diagnostic test (RDT) SD Bioline Malaria Ag P.F (Alere _TM_) and if positive were considered clinical malaria cases and treated according to national guidelines. An additional blood sample (300 µL) was collected by finger prick into an EDTA microtainer tube for molecular detection and quantification of *P. falciparum* gametocytes. For only the children in the SMC group, information on SMC adherence was collected as reported by care givers. In addition, each child’s SMC administration card was reviewed and administered doses as documented was transcribed on the study case report form and used to assess participants’ adherence to the treatment.

### Sample processing

Blood samples collected in EDTA tubes during both study visits were stored in a cool box and transported to the laboratory within a maximum of 6 h after collection to maintain ribonucleic acid (RNA) stability [24]; 70 µl of whole blood were immediately transferred into 350 µl of RNAprotect Cell Reagent (Qigen, Hilden, Germany) and stored at -70ºC. Laboratory staff were blinded to the study group of each sample during analysis. Each set of a participant’s paired samples (i.e. sample for the same participant collected before and after SMC administration) were analysed for gametocytes in the same run to avoid variation in laboratory procedures.

*Plasmodium falciparum* diagnosis was performed at screening by PCR. Parasite deoxyribonucleic acid (DNA) was extracted from DBS using the QiaAmp DNA minikit (Qiagen, Germany). The *var* gene acidic terminal sequence (*var*ATS) quantitative PCR was used to detect multi-copy genomic sequences of infections [25]. Briefly, genomic DNA of the parasite was amplified in 20 µl reaction containing 1 × Taqman mastermix (Life Technologies, United Kingdom) and run in CFX96 Touch™ real-time PCR detection system (Bio-Rad, United Kingdom). The starting quantity values of the parasite samples were estimated against laboratory grown *P. falciparum* 3D7 standard control (with medonic read of 3.74 × 10^6^ erythrocytes/µl and thin film parasitaemia of 1197 parasites/µl of blood).

For *P. falciparum* gametocytes detection for the samples collected before and after SMC treatment, RNA was extracted using Qiagen’s RNeasy® Mini kit according to the manufacturer’s recommendations. *Pfs*25 Quantitative Nucleic Acid Sequence-based Amplification (QT-NASBA) real-time PCR was performed on the extracted mRNAs using the following primers (forward primer: 5′-GACTGTAAATAAACCATGTGGAGA-3′; reverse primer 5′-AATTCTAATACGACTCACTATAGGGAGAAGGCATTTACCGTTACCACAAGTTA-3′) and PCR conditions **(**pre-heat at 65 °C; incubate at 65 °C for 2 min then 41 °C for 2 min; a further 41 °C for 46 s incubation post enzyme introduction). Gametocytaemia was determined using fluorescence amplification time-points in correlation with the standard dilution series that was included in each run [22].

### Statistical analysis

Sample size was calculated assuming baseline gametocyte prevalence of 50% in both the SMC and comparison groups [22]. A sample size of 116 evaluable participants in total, 58 per group, would have 80% power to detect a 25% difference (increase) in gametocyte prevalence between groups at the 5% significance level.

Statistical analysis was performed using STATA software version 16.0 (Stata Corp, College Station, Texas, USA). Descriptive statistics are presented for continuous variables (median (IQR)) and proportions for categorical variables; point estimates are presented with 95% confidence intervals. Analysis was restricted to study participants who remained asymptomatic from enrolment to the time of post-treatment blood sampling. Gametocyte prevalence in children that received at least the first dose of SMC treatment (SP plus AQ) and in those that adhered to all 3-day doses of SMC treatment (SP plus AQ on day 1 and AQ alone on days 2 and 3) was each compared with gametocyte prevalence in the comparison group. Difference in prevalence between the two groups was assessed using the chi-squared test. Conditional logistic regression, conditional on individuals, was used to predict the odds of gametocyte carriage before and after treatment within each group.

### Ethical consideration

This study was approved by the Gambia Government/MRC Joint Ethics Committee (SCC 1563). Children’s parents or legal representative provided written informed consent prior to screening and study participation.

## Results

**Fig. 1:**
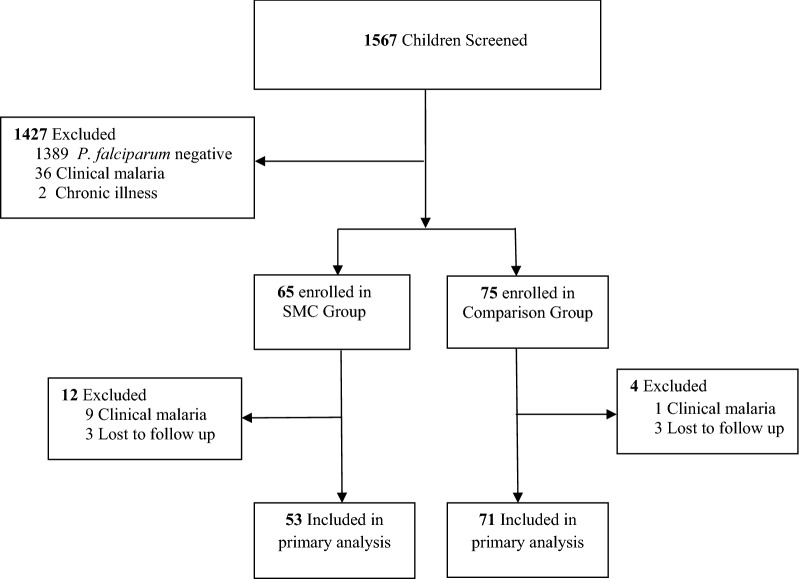
Study Diagram

A total of 1,567 children were screened for malaria infection at the beginning of two successive transmission seasons (2017 and 2018); 871 children aged 2 to < 5 years (SMC group) and 696 aged 5 to 8 years (comparison group). Among these, 65 (7.5%) children in the SMC group and 75 (10.8%) in the comparison group had asymptomatic *P. falciparum* infection (Fig. 1). Baseline characteristics are as shown in Table 1. Gametocyte prevalence before SMC treatment was 10.7% (7/65) and 13.3% (10/75) in the SMC and comparison groups respectively. Median interval (days) between receiving SMC treatment and blood sampling for assessment of post-treatment gametocytaemia was 13 days (IQR 12, 13). At 13 days post-treatment, there was no difference in gametocyte prevalence between children in the SMC group who received at least the first dose of SMC treatment (SP and AQ) (9.4%, 5/53) and those in the comparison group (12.7%, 9/71), (*p* = 0.57). Although gametocyte prevalence was higher in children who adhered to all 3-day doses of SMC treatment (15.6%, 5/32), this was not significantly different with gametocyte prevalence of those in the comparison group (*p* = 0.68) (Table 2). Within the SMC group, gametocyte carriage tended to be lower after SMC treatment in children that received at least the first dose of SMC treatment, but this was not statistically significant (OR:0.6, 95% CI: 0.14 to 2.51, *p* = 0.48). Likewise, there was no change in odds of gametocyte carriage after treatment among children that adhered to all 3-day doses of SMC treatment (OR 1.0, 95% 0.20–4.95, *p* = 1.0).

**Table 1:** Baseline characteristics of study participants (N = 140)

Characteristic	Group	
	SMC (n = 65)	Comparison (n = 75)
Gender (female), n (%)	31 (47.7)	35 (46.7)
Age (years), median (IQR)	3.6 (2.7–4.5)	6.5 (5.2–7.5)
Weight (kg), median (IQR)	12.8 (11.0–14.2)	18.0 (15.5–20.6)
Haemoglobin (g/dL), median (IQR)	10.9 (9.9–12.0)	11.1 (10.0–12.2)
Asexual parasite density (per µL), geometric mean (95%CI)	0.53 (0.16–1.78)	0.59 (0.15–2.36)

**Table 2:** Differences in gametocyte carriage before and after SMC treatment

Measure	Group		p value
	SMC	Comparison	
Percentage of gametocyte carriers before SMC treatment	7/65 (10.7%)	10/75 (13.3%)	0.64*
Percentage of gametocyte carriers after SMC treatment			
Received at least first dose of treatment	5/53 (9.4%)	9/71 (12.7%)	0.57*
Received all 3-day doses of treatment	5/32 (15.6%)	9/71 (12.7%)	0.68*
Odds ratio comparing gametocyte carriage before vs after SMC treatment. OR (95% CI)			
Received at least first dose of treatment	0.6 (0.14–2.51)		0.48^∞^
Received all 3-day doses of treatment	1.0 (0.20–4.95)		1.00^∞^
		0.9 (0.34–2.30)	0.80^∞^

* Chi- squared test used to assess between-group difference in proportion of gametocyte carriers

^∞^Conditional logistic regression used to determine within-group odds of gametocyte carriage

**Fig. 2:**
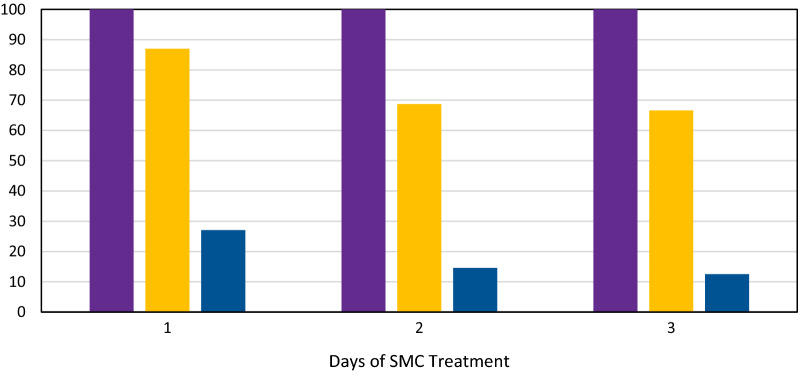
Percentage of children that adhered to SMC treatment as reported by care giver (purple), as documented on the SMC distribution card (amber) and those that vomited administered dose shortly after administration (blue)

Adherence to SMC treatment and occurrence of vomiting after treatment was assessed in 90% (48/53) of children in the SMC group (Fig. 2). For all of them, the caregiver reported to have administered the remaining 2 days of treatment. When this information was cross-checked with the SMC drug administration card, only 68.7% (33/48) and 66.6% (32/48) had the doses of the second and third days documented on their cards, respectively. A total of 27.1% (13/48) of children were reported to have vomited the dose for the first day shortly after administration while 14.6% (7/48) and 12.5% (6/48) did so for the doses second and third days, respectively.

## Discussion

SP and AQ, the anti-malarial drugs deployed for the SMC intervention, are associated with marked increase in gametocytaemia when used to treat clinical cases of *P. falciparum* malaria [10, 12, 14]. Whether the administration of these drugs would increase prevalence of gametocytes among children with asymptomatic *P. falciparum* infections receiving SMC was investigated; no evidence of increased gametocyte prevalence was found.

Only a few studies have assessed the emergence of post-treatment gametocytes following treatment of asymptomatic *P. falciparum* infections and to our knowledge, no study has addressed this question in the context of the SMC. Dunyo et al. [26]*.* in a randomized controlled trial (RCT) assessed whether SP increased gametocyte carriage following treatment of asymptomatic *P. falciparum* carriers that had asexual parasite densities > 20 parasites per micro litre but found no evidence of increased gametocyte prevalence or density. However, in that trial, microscopy was used to detect gametocytes and an increase in gametocytaemia may have been missed since emerging gametocytes typically circulate at or below the microscopic detection threshold [15]. In addition, considering the study by Dunyo et al*.* was a RCT, participants would have received the full treatment dose, with little or no risk of subcurative dosing that could trigger gametocyte production [27]. Conversely, sub-optimal dosage is probable in the context of standard SMC implementation wherein 2 out of the 3 daily doses are administered by caregivers [19, 20] and vomiting of administered doses shortly after administration is common [28]. However, even with the risk of sub-optimal dosaging and the use of sensitive molecular methods for detection of gametocytes in the current study, there was no evidence of increased gametocyte carriage following SMC in children that received either at least the first dose of SMC treatment or all 3-day doses of SMC treatment confirming findings by Dunyo et al*.* [26]. Of note however is that individuals enrolled in the current study had very low parasite densities (geometric mean < 1 parasite/uL), representative of typical asymptomatic parasite carriers in low-endemic settings [29]. However, whilst the current study thus had much lower gametocyte prevalence than typically reported in infections with higher parasite densities [29], it has the advantage of including participants that reflect infected individuals among the actual target population of SMC.

In the current study population with low parasite density and low baseline gametocyte prevalence, sensitive molecular gametocyte detection methods did not reveal a significant effect of SMC (neither increase nor decrease) on gametocyte carriage shortly after treatment. Perhaps over longer-periods of follow-up, the clearance of asexual parasites, the precursors of gametocytes, may result in lower gametocyte carriage. This result could be enhanced if gametocyte-clearing drugs would be added to the SMC regimen, reducing gametocytes persistence after treatment that was also evident in the current study [18]. Such strategy might confer benefits for malaria transmission in the community.

Adherence to SMC administration as reported by caregivers was 100%, similar to what has been reported from Mali [30]. However, when comparing reported adherence to the information on the SMC distribution card (documented adherence), the second and third doses were documented in only two thirds of study participants (Fig. 2), suggesting either caregivers’ over-reporting of adherence during the interviews or incomplete recording. In a malaria chemoprevention trial in Uganda, drug levels were detectable in only 52% of cases despite caregivers having reported administering all assigned doses [20], suggesting over reporting of adherence. Similarly, a study on SMC uptake in Ghana concluded that up to 20% of children did not receive their second and third days treatments based on the number of tablets found at their homes during household visits [19]. Reported adherence by caregivers is, therefore, unreliable; possibly all SMC treatment doses should be directly observed by health workers or by trained village health workers, even though this approach could pose logistical challenges.

Vomiting occurred more often than expected [28], 27% of children vomited the first treatment dose; although the percentage was lower for the second or third dose, this could be due to under-reporting by the caregivers. Low tolerability may lead to low adherence, resulting in sub-curative doses in malaria infected individuals, with potential risks of selecting for drug resistant parasites [31], emergence of post-treatment gametocytaemia [15] and onward malaria transmission [32]. Sweetened dispersible tablets resulted in higher tolerability in Senegal [33] and should perhaps be adopted as the standard formulation for SMC. Furthermore, provision should be made to compensate for vomited doses, particularly those administered by caregivers at home.

There are some limitations to this study that should be considered. Baseline gametocyte prevalence was lower than expected and this may have affected the power of the study to find a significant difference between SMC and comparison groups. However, there was little difference in gametocyte prevalence before and after treatment in the SMC group (10.7% *versus* 9.4%) similar to what was observed in the control group (13.3% *versus* 12.7%), suggesting that SMC is not associated with increased gametocyte carriage in the current study. Secondly, the fitness of gametocytes was not assessed as anti-malarial drugs may sterilize gametocytes before clearing them from circulation [34] since gametocyte transmissibility is ultimately more relevant for public health than gametocyte presence. Thirdly, drug concentrations were not measured and thus it is not possible to carry out a more detailed analysis on the effect of suboptimal dosing on post-treatment gametocytaemia. Finally, gametocytaemia was measured only at one time point after SMC treatment and this did not allow for plotting the dynamics of circulating gametocytes over a longer period.

## Conclusion

In this low transmission setting, there is no evidence that SMC administered to asymptomatic *P. falciparum* infected children is associated with higher prevalence of gametocytes. This therefore argues against a possible increase in transmission after SMC campaigns. However, these findings cannot be generalizable to other settings since factors that could determine the emergence of post-treatment gametocytes, notably asexual parasite density [15] and drug resistance [35], vary across different transmission settings. As SMC is delivered to millions of children across different transmission settings of the Sahel, there is the need to investigate this question in other settings ideally with direct assessments of transmission potential of gametocytes to mosquitoes.

## Data Availability

The data used or analysed in this study are available from the corresponding author on reasonable request with approval from the Gambia Government/MRC Joint Ethics Committee.

## Abbreviations

AQ: Amodiaquine
CQ: Chloroquine
DBS: Dried blood spot
DNA: Deoxyribonucleic acid
EDTA: Ethylene diamine tetraacetic acid
HDSS: Health and Demographic Surveillance System
IPTp: Intermittent preventive treatment during pregnancy
IRS: Indoor residual spraying
ITN: Insecticide treated nets
MRC: Medical Research Council
PQ: Piperaquine
PCR: Polymerase chain reaction
QT-NASBA: Quantitative nucleic acid sequence-based amplification
RCT: Randomized controlled trial
RDT: Rapid diagnostic test
RNA: Ribonucleic acid
SMC: seasonal malaria chemoprevention
SP: Sulfadoxine-pyrimethamine
*var*ATS: *var* Gene acidic terminal sequence
WHO: World Health Organization
